# Hypoxia facilitates neurogenic dural plasma protein extravasation in mice: a novel animal model for migraine pathophysiology

**DOI:** 10.1038/srep17845

**Published:** 2015-12-08

**Authors:** Anika Hunfeld, Daniel Segelcke, Ingo Bäcker, Badreddine Mecheri, Kathrin Hemmer, Elisabeth Dlugosch, Michael Andriske, Frank Paris, Xinran Zhu, Hermann Lübbert

**Affiliations:** 1 Institute of Animal Physiology, Ruhr-University Bochum, 44780 Bochum, Germany

## Abstract

Migraine animal models generally mimic the onset of attacks and acute treatment processes. A guinea pig model used the application of meta-chlorophenylpiperazine (mCPP) to trigger immediate dural plasma protein extravasation (PPE) mediated by 5-HT_2B_ receptors. This model has predictive value for antimigraine drugs but cannot explain the delayed onset of efficacy of 5-HT_2B_ receptor antagonists when clinically used for migraine prophylaxis. We found that mCPP failed to induce dural PPE in mice. Considering the role 5-HT_2B_ receptors play in hypoxia-induced pulmonary vessel muscularization, we were encouraged to keep mice under hypoxic conditions and tested whether this treatment will render them susceptible to mCPP-induced dural PPE. Following four-week of hypoxia, PPE, associated with increased transendothelial transport, was induced by mCPP. The effect was blocked by sumatriptan. Chronic application of 5-HT_2B_ receptor or nitric oxide synthase blockers during hypoxia prevented the development of susceptibility. Here we present a migraine model that distinguishes between a migraine-like state (hypoxic mice) and normal, normoxic mice and mimics processes that are related to chronic activation of 5-HT_2B_ receptors under hypoxia. It seems striking, that chronic endogenous activation of 5-HT_2B_ receptors is crucial for the sensitization since 5-HT_2B_ receptor antagonists have strong, albeit delayed migraine prophylactic efficacy.

Despite the high prevalence of migraine, physiological mechanisms predisposing patients to the pain attacks are largely unknown. A major challenge has been the development of animal models, which not only simulate aspects of acute migraine attacks, but rather address the underlying migraine condition that facilitates the onset of attacks. Available animal models mainly address cortical spreading depression, trigeminovascular interactions, central control mechanisms, dural vasodilation and plasma protein extravasation (PPE)^1^,^2^. It is widely accepted that migraine pain is transmitted by perivascular trigeminal nerve fibres originating from neurons in the trigeminal ganglion^3^. Electrical stimulation of the trigeminal ganglion resulted in a marked increase in PPE in the ipsilateral dura mater of rats, guinea pigs and mice^4^,^5^,^6^. The electrical stimulation released neuropeptides from perivascular axons, consequently triggering PPE, vasodilation, and mast cell degranulation, causing the release of further proinflammatory compounds^4^,^7^,^8^,^9^,^10^. This approach to induce neurogenic inflammation in the meninges explained the therapeutic relevance of serotonin(5-HT)_1B/1D/1F_ receptors expressed on inter- and intracranial blood vessels and the peripheral endings of trigeminovascular afferents or trigeminal ganglion^11^,^12^. Agonists on 5-HT_1B/1D/1F_ receptors were shown to interfere with neurogenic inflammation^11^,^13^ and the 5-HT_1B/1D_ agonist sumatriptan has been frequently used to interrupt ongoing migraine attacks in the clinic ever since. The approach to use highly selective 5-HT_1F_ agonists displays a further promising target for acute therapy^14^,^15^.

Instead of the electrical stimulation, application of the 5-HT_2B/2C_ receptor agonist meta-chlorophenylpiperazine (mCPP) also induced dural PPE in guinea pigs^6^,^16^. The involvement of this group of receptors, particularly the 5-HT_2B_ receptor, in the onset of migraine attacks had been deduced from various observations, such as the prophylactic action of compounds antagonizing the receptor, such as methysergide, pizotifen or cyproheptadine^17^, as well as the receptor’s endothelial localization^18^ and nitric oxide (NO) coupling^19^,^20^. mCPP-induced PPE was indeed inhibited by selective 5-HT_2B_ receptor antagonists or the nitric oxide synthase (NOS) inhibitor L-NAME in the guinea pig model, indicating the involvement of 5-HT_2B_ receptor activation and NO formation^6^. However, the induction of PPE in guinea pigs and its inhibition by 5-HT_2B_ receptor antagonists are immediate effects occurring within minutes, while the prophylactic action of this class of migraine preventive drugs is a delayed response requiring several weeks of treatment before the frequency and intensities of migraine attacks decline^14^. Here we describe a mouse model that may mimic some of the chronic changes that render migraineurs susceptible to migraine attacks.

## Results

### mCPP-induced dural PPE occurs in mice only after chronic hypoxia

**Figure 1:**
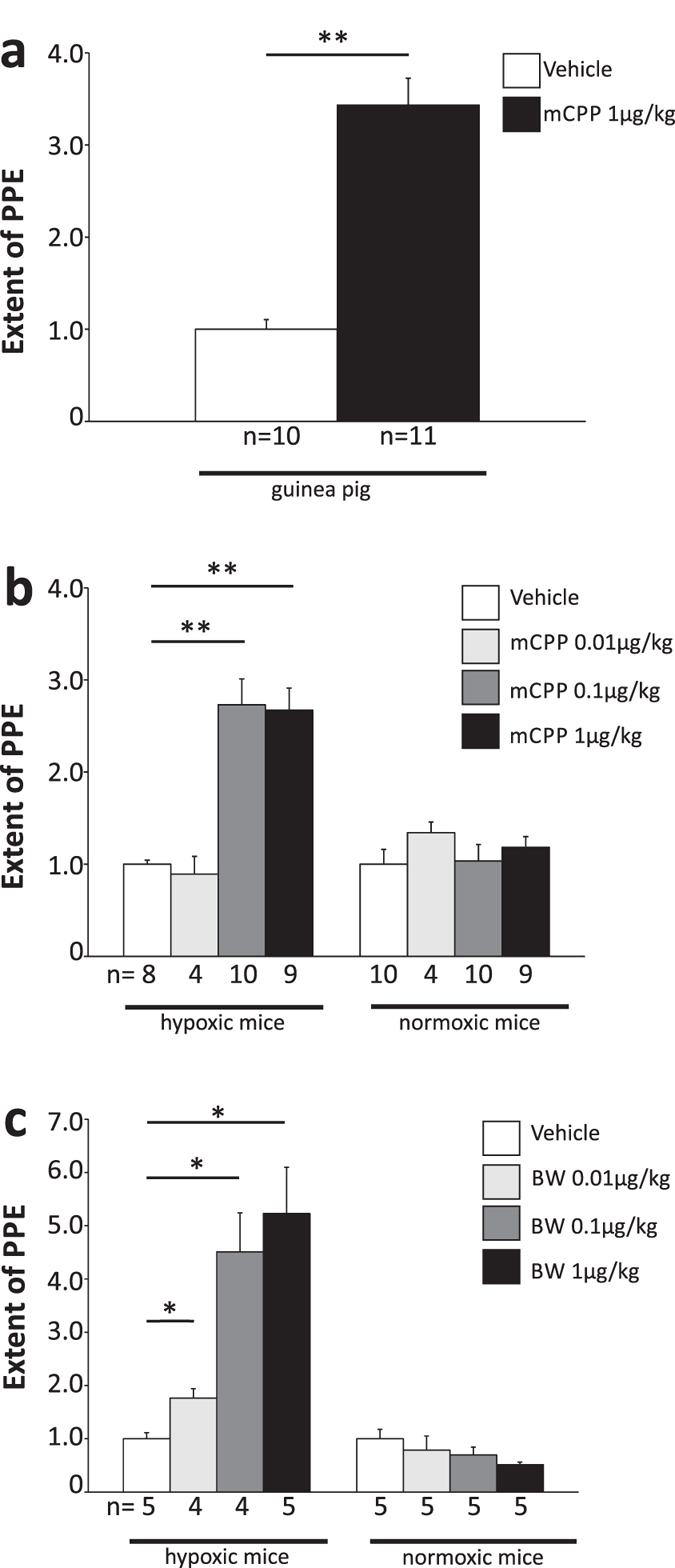
Hypoxia-induced effects in the dura mater of rodents. (**a**) The 5-HT_2B/2C_ agonist mCPP induced PPE in the dura mater of the guinea pig. (**b**) mCPP dose-dependently induced PPE in the dura mater of hypoxic but not normoxic mice. (**c**) The more specific 5-HT_2B_ agonist BW 723C86 (BW) dose-dependently induced PPE in the dura mater of hypoxic but not normoxic mice. Data normalized to negative control (vehicle). All applications i.v. Mean ± SEM. Statistics: *p < 0.01, **p < 0.001; Rank Sum Test (**a**), multiple pairwise Rank Sum Test comparison with alpha-correction (**b,c**).

To further characterize the mCPP-induced PPE described in guinea pigs, we attempted to transfer the model to mice and rats, and found that doses of mCPP that were effective in guinea pigs (Fig. 1a) did not induce dural PPE in normoxic mice (Fig. 1b) or rats (data not shown). The affinity of mCPP towards human and rat 5-HT_2B_ receptors is almost identical, in the nanomolar range^21^. We thus did not expect big differences in affinity towards murine 5-HT_2B_ receptors. In addition, differences in the drug’s bioavailability or pharmacokinetic properties alone cannot explain the aforementioned effect since mCPP was applied intravenously and the effect occurs within 17 minutes after drug application in guinea pigs, barely sufficient to be strongly affected by species differences in the drug’s metabolism or pharmacokinetics.

It is well established that chronic hypoxia treatment of mice or rats triggers muscularization of lung arterioles, and hypoxia treatment for several weeks is thus employed as a model for pulmonary hypertension^22^. Chronic 5-HT_2B_ receptor activation seems to be involved in this phenomenon since a lack of receptor activation during hypoxia prevents the hypermuscularization^23^. We established this model in mice, verified the hypermuscularization in lung blood vessels, and, following four weeks of hypoxia with 10% oxygen, found that hypoxic mice had developed a sensitivity towards mCPP-induced PPE (Fig. 1b). In hypoxic mice, the maximal dural PPE was triggered with 0.1 μg/kg of mCPP, and no further increase was seen with 1 μg/kg of mCPP. Both concentrations, and even 250 μg/kg of mCPP (data not shown), had no effect in normoxic mice (Fig. 1b).

### mCPP-induced dural PPE in hypoxic mice is similar to that in guinea pigs

**Figure 2:**
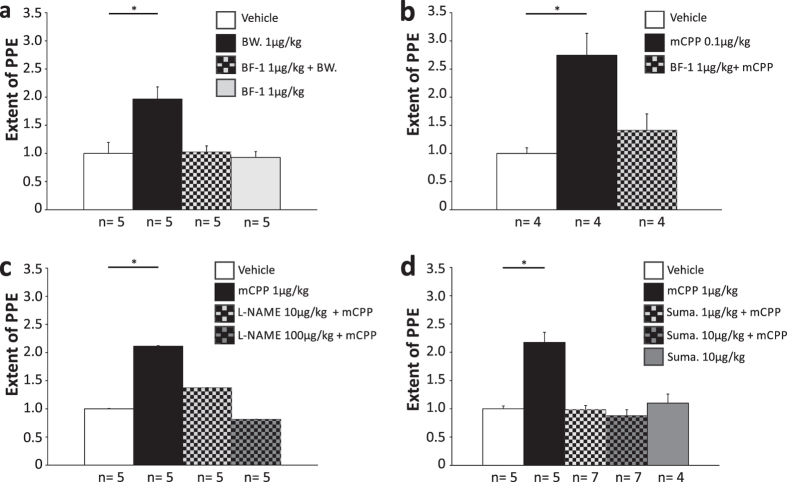
Acute blockage of the PPE induced with mCPP and BW 723C86 in hypoxic mice. (**a**) The BW 723C86 (BW)-induced PPE in hypoxic mice was blocked with BF-1. (**b**) The mCPP-induced PPE in hypoxic mice was blocked with BF-1. (**c**) The mCPP-induced PPE in hypoxic mice was blocked with L-NAME. (**d**) The mCPP-induced PPE in hypoxic mice was blocked with sumatriptan (suma). Data normalized to negative control (vehicle). All applications i.v. Mean ± SEM. Statistics: *p < 0.05, One-Way ANOVA and Posthoc test (Bonferroni t-test).

To compare the properties of mCPP-induced dural PPE in hypoxic mice with that in guinea pigs, some characteristic pharmacological properties of the PPE were investigated. To verify the involvement of 5-HT_2B_ receptors, dural PPE was triggered with BW 723C86, a 5-HT_2B_ receptor agonist that is more selective than mCPP^24^. Similar to mCPP, 0.1 and 1 μg/kg BW 723C86 induced dural PPE in mice after four weeks hypoxia, but not in normoxic animals (Fig. 1c). Furthermore, BF-1, a selective and high-affinity 5-HT_2B_ receptor antagonist completely inhibited BW 723C86- or mCPP-induced extravasation in hypoxic mice (Fig. 2a,b). BF-1 was previously shown to inhibit mCPP-induced dural PPE in guinea pigs and was effective at 100-fold lower concentrations than those required for methysergide or pizotifen^16^. These results confirm that the dural PPE induced by mCPP or BW 723C86 in hypoxic mice is mediated by the activation of 5-HT_2B_ receptors.

In guinea pigs, the mCPP-induced dural PPE was dependent on 5-HT_2B_ receptor-mediated NO release and the activation of trigeminal fibres^6^. To investigate whether NO release was necessary for the production of mCPP-induced dural PPE in hypoxic mice, animals were pretreated with L-NAME, a non-specific NOS inhibitor, at a dose that by itself has no effect on PPE in the dura mater as it was seen in the experiments performed in the guinea pig^6^. In these experiments, L-NAME inhibited the dural PPE in hypoxic mice (Fig. 2c).

To confirm that the formation of mCPP-induced dural PPE in hypoxic mice was also mediated by activating the trigeminal pathway, the 5-HT_1B/D_ receptor agonist sumatriptan was used. This anti-migraine drug blocked dural PPE in hypoxic mice (Fig. 2d). Together these results demonstrate that the mCPP-induced dural PPE in hypoxic mice displayed key characteristics of migraine pain attacks, like the dural PPE triggered by mCPP in guinea pigs^6^,^16^.

### mCPP-induced dural PPE in hypoxic mice is not caused by an acute response to hypoxia

**Figure 3:**
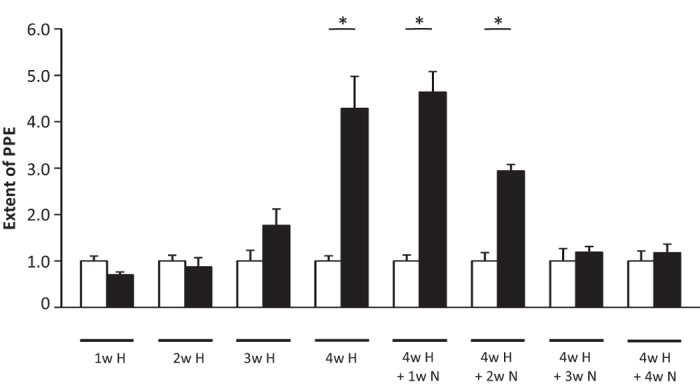
Reversibility of the hypoxia-induced effect by subsequent normoxic treatment. It was possible to induce dural PPE by intravenous application of mCPP 1 μg/kg after at least four weeks of hypoxia. This effect lasted for two weeks of subsequent normoxic treatment. Data normalized to negative control (vehicle). 1w H = one week hypoxia. 4w H + 1w N = four weeks hypoxia + one week normoxia. White: Vehicle, black: mCPP 1 μg/kg. All applications i.v. Mean ± SEM. n = 5 per group. Statistics: *p < 0.01, Multiple pairwise comparison with alpha-correction (Rank Sum Test).

To obtain further insight into the nature of this effect, we examined whether the elevated susceptibility towards dural PPE is an acute or a transient response to hypoxia. In the next set of experiments, we investigated the minimal duration of the hypoxia treatment required for mCPP-induced dural PPE in mice to occur, and found that it took three weeks of hypoxia before an elevation of mCPP-induced dural PPE becomes visible (Fig. 3). After four weeks of hypoxia, the dural PPE reached its maximum (Fig. 3), which could not be enhanced further by extending the duration of hypoxia (data not shown).

Chronic hypoxia causes remodelling of blood vessels in the lung, associated with increased vascular muscularization, serving as an animal model for pulmonary hypertension^22^,^23^. Pulmonary vessel remodelling persists, after animals returned to normoxic conditions, for at least several weeks or until death^25^. To determine how long the mCPP-induced dural PPE in hypoxic mice persisted after hypoxia, we observed our hypoxic mice after they returned to normoxic conditions for several weeks. The susceptibility to mCPP-induced dural PPE, occurring after hypoxia, was still apparent after two weeks but declined within three weeks after hypoxia (Fig. 3). In littermates, enhanced muscularization in lung blood vessels was stable for at least 8 weeks without any tendency to return to normal levels (data not shown). These observations indicate that the enhanced dural mCPP response in mice, caused by chronic hypoxia, was associated with changes that were reversible, but lasted for several weeks.

### The sensitivity towards PPE in the dura mater following hypoxia is related to enhanced transcytosis rather than hypermuscularization

**Figure 4:**
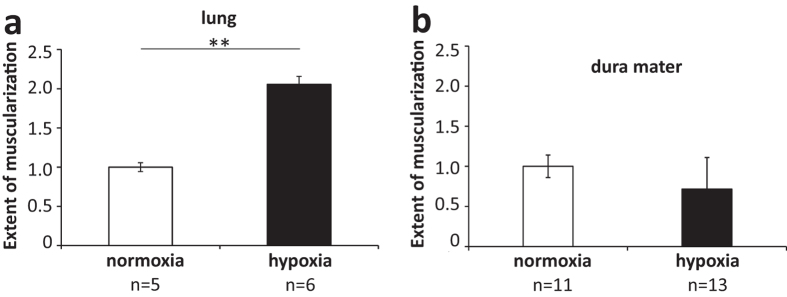
Hypoxia-induced vascular remodelling occurred in the lung but not in dura mater. After four weeks of hypoxia treatment, mice displayed a significantly increased number of arterial blood vessels with a diameter 25 μm ≤ Ø ≤ 50 μm in the lung (**a**). In dura mater, the length of muscularized arterioles with a diameter Ø ≥ 20 μm was equivalent between hypoxic and normoxic mice (**b**). Data normalized to negative control (normoxia). Mean ± SEM. Statistics: **p < 0.01, Rank Sum Test.

**Figure 5:**
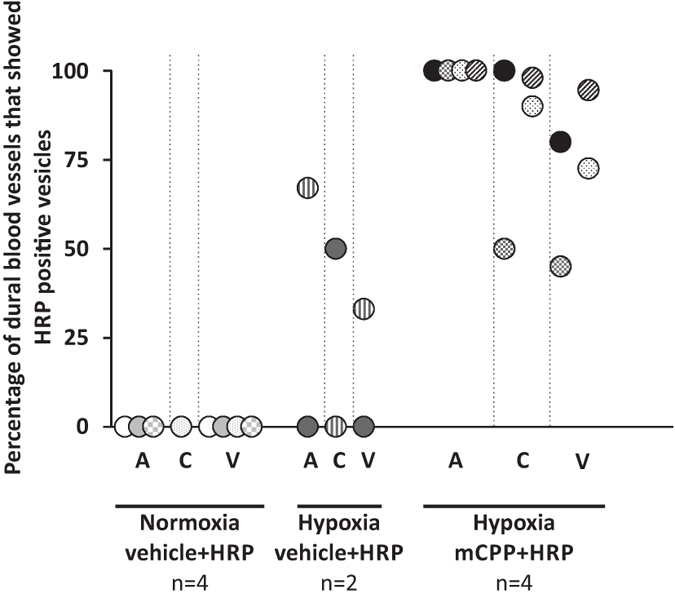
Quantification of HRP-positive endothelial vesicles in dural blood vessels. The percentage of dural arterioles (A), capillaries (C) and venules (V) that showed HRP-positive vesicles in the endothelium is indicated for individual mice treated with vehicle + HRP (n = 4 normoxic and n = 2 hypoxic mice) and for mice treated with mCPP 1 μg/kg + HRP (n = 4 hypoxic mice). HRP 200 mg/kg. Data points of same colour/ pattern represent data for one individual mouse. All applications i.v.

Even though the chronic changes leading to enhanced susceptibility towards PPE in the dura mater may be similar to those seen in the lung, changes in the dura mater were not quite as long lasting as the hypermuscularization of lung blood vessels and may thus be different in nature. To investigate similarities to the changes in the lung in further detail, we examined if enhanced muscularization of blood vessels occurred in the dura mater. While we quantified increased muscularization in lung blood vessels after four-week hypoxia (Fig. 4a), we did not detect similar changes in the dura mater (Fig. 4b). This tempted us to look at hypoxia-induced changes of dural blood vessels by electron microscopy. For this set of experiments, mice were treated with vehicle or mCPP combined with horseradish peroxidase (HRP, 200 mg/kg). The selected dose of mCPP fully induced PPE when measured with the tracer Evans Blue (Fig. 1b). We investigated cross sections of dural blood vessels and distinguished three major branches of the dural vasculature by morphological parameters (see Supplementary Methods 1) and investigated ultrastructural deposits of the tracer in cells forming the vasculature (representative pictures are shown in Supplementary Figure S1). To quantify the occurrence of HRP-positive endothelial vesicles in the treatment groups, each photographed blood vessel was investigated whether it showed this feature or not. Quantification results are shown in Fig. 5. Each data point represents the percentage of blood vessels that were positive for the aforementioned feature in an individual mouse, e.g., a mCPP-treated hypoxic mouse expressed HRP-positive vesicles in endothelial cells in 100% of investigated arterioles, in 100% of investigated capillaries and in 80% of investigated venules (black data points, Fig. 5). HRP-positive vesicles occurred more frequently in all three types of vasculature after injecting mCPP when compared with vehicle. Interestingly, vehicle-treated normoxic mice did not show HRP-positive endothelial vesicles (Fig. 5). Thus, hypoxia treatment by itself appeared to induce elevated levels of transcytosis in the dura mater and stimulation with mCPP then further triggered massive transcytosis.

**Figure 6:**
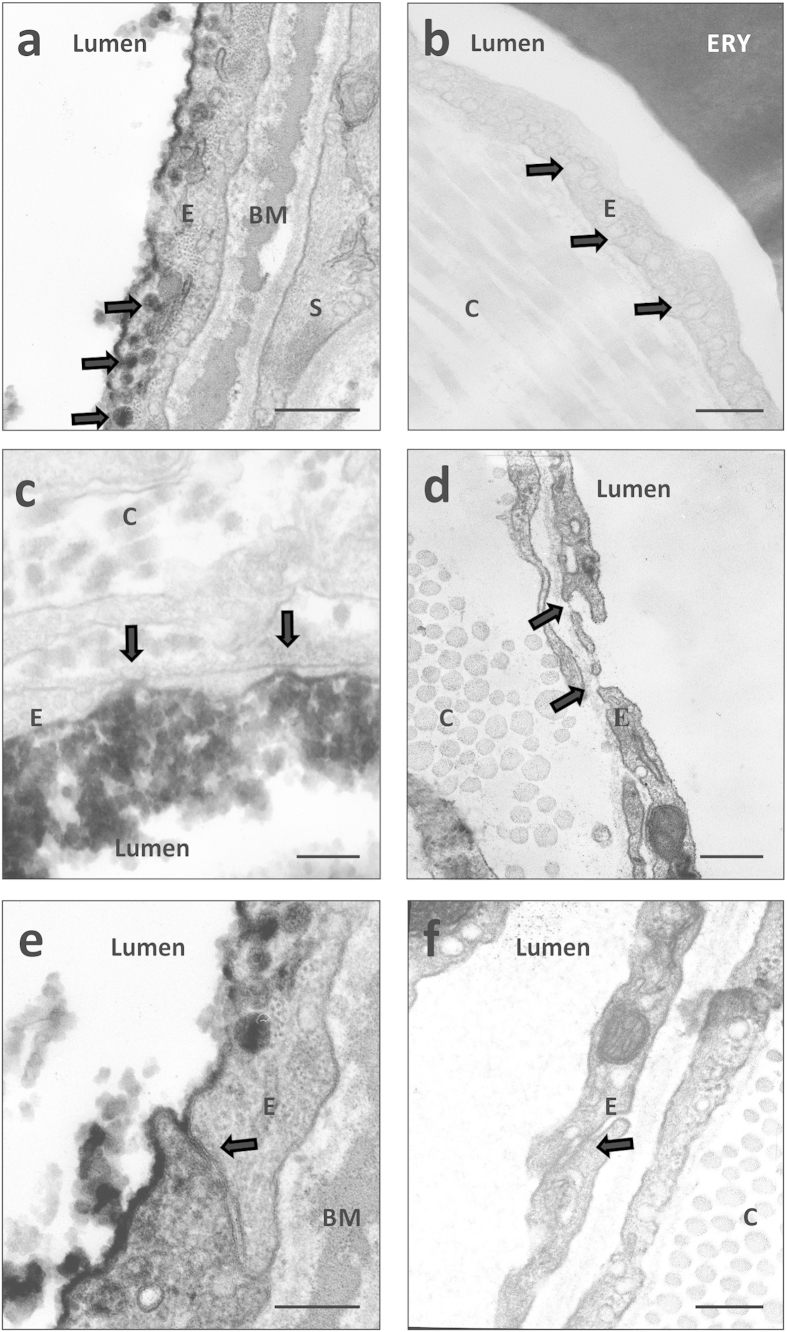
Detailed depiction of HRP-positive and negative ultrastructures confirmed HRP extravasation via a transcellular pathway. Higher magnifications of animals that received most extensive treatment (mCPP + HRP) and animals that received fewest treatment (vehicle + vehicle) confirmed that HRP-positive ultrastructures (such as HRP-positive endothelial vesicles (**a**, arrows) occurred exclusively in animals that received the dye but not in controls (**b**, arrows). It was further confirmed, that HRP did not penetrate via endothelial fenestrae (**c**, arrow) and that the tracer stopped at the level of interendothelial tight junctions (**e**, arrow). There was no damage in fenestrae structure (**D**, arrow) or tight junction integrity (**f**, arrow). Scale bars: **a**, **b**, **e**, **f**: 1 μm; **c**, **d**: 0.25 μm.

A more detailed look at the endothelial ultrastructure illustrated, that PPE in this model occurred via a transcellular pathway: HRP-filled vesicles pinched off the endothelial cell lining (arrows, Fig. 6a). Perivascular macrophages then took up the dye in the periphery of the vasculature (Supplementary Figure S1a, c, d, f). Endothelial fenestrae remained intact (arrows, Fig. 6d) and retained the tracer HRP when administered (arrows, Fig. 6c). In addition, interendothelial tight junctions were intact (arrow, Fig. 6f) and stopped the extravasation of the tracer (arrow, Fig. 6e). The presence of intact fenestrae and tight junctions further indicated that the observed findings did not represent an artefact due to the systemic perfusion of the animals. HRP-positive structures did not occur in preparations of mice that received vehicle instead of HRP and were thus indeed due to HRP (Fig. 6b, d and f).

Altogether, these findings demonstrate that PPE in this model occurred via a transcellular pathway and that hypoxia treatment very likely increased the susceptibility towards this process.

### Formation of hypoxia-induced susceptibility to dural PPE in response to mCPP in mice is 5-HT_2B_ receptor-dependent

**Figure 7:**
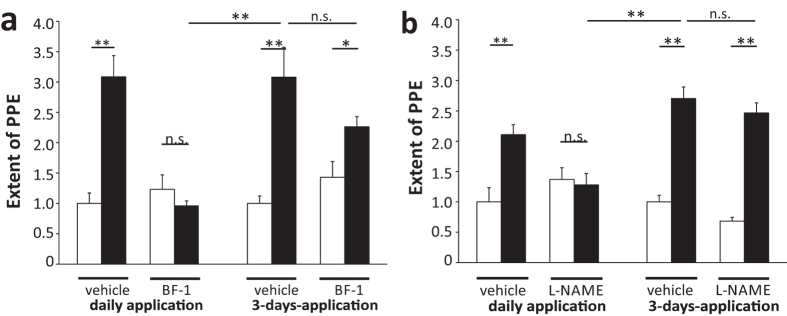
Chronic blockage of the 5-HT_2B_ receptor and NOS during hypoxia. (**a**) The mCPP-induced PPE in hypoxic mice was blocked by daily BF-1 applications (20 mg/kg i.p.) during the sensitization phase. The application of BF-1 on the last three days of the hypoxic treatment was not sufficient to block the mCPP-induced PPE significantly. (**b**) The mCPP-induced PPE in hypoxic mice was blocked by daily L-NAME applications (20 mg/kg i.p.) during the sensitization phase. The application of L-NAME on the last three days of the hypoxic treatment was not sufficient to block the mCPP-induced PPE significantly. White: Vehicle, black: mCPP 1 μg/kg. n = 5 per group. Data normalized to negative control (vehicle + vehicle). Mean ± SEM. Statistics: *p < 0.05, **p < 0.01, n.s. - not significant, Multiple pairwise comparison with alpha-correction (Rank Sum Test).

To start unravelling how hypoxia induced changes that lead to increased susceptibility towards mCPP and elevated levels of endothelial transcytosis in dural blood vessels, we tested whether this process required 5-HT_2B_ receptor activation, in analogy to the hypermuscularization in the lung^23^. We therefore blocked 5-HT_2B_ receptors during the four-week hypoxia by daily injections of the highly selective 5-HT_2B_ receptor antagonist BF-1 (20 mg/kg i.p). Subsequently, mice were kept under normoxic conditions for a week to ensure complete washout of BF-1 before they were challenged with mCPP. The chronic application of BF-1 during the hypoxic treatment completely prevented mCPP-induced dural PPE in mice (Fig. 7a). To ascertain that this inhibition was not caused by residual BF-1 in the mice, we tested mCPP-induced dural PPE in hypoxic mice that had received BF-1 injections only during the last three days of hypoxia. These injections did not prevent mCPP-induced dural PPE one week after hypoxia (Fig. 7a). Thus, the effect could not be caused by residual BF-1 interfering with the acute response to mCPP. When L-NAME was administered daily during hypoxia, we found that the mCPP-induced PPE was prevented as well and that this effect was not caused by residual L-NAME since L-NAME injections on the three last days of hypoxia were not sufficient to block the mCPP-induced PPE (Fig. 7b).

We conclude that chronic activation of 5-HT_2B_ receptors and NOS, which occurred physiologically during the hypoxia treatment, was necessary to develop the increased susceptibility to mCPP-induced dural PPE in mice.

## Discussion

mCPP has been reported to trigger severe migraine attacks or migraine-like symptoms in human subjects with a history of migraine^26^,^27^. It has consequently been used to trigger dural neurogenic inflammation in guinea pigs^6^,^16^, adding a different experimental approach to the previously used electrical stimulation of the trigeminal nerve in rodent models^4^. In guinea pigs, mCPP has been shown to induce plasma protein extravasation via the stimulation of 5-HT_2B_ receptors, subsequent NO production and activation of trigeminal nerve endings^6^.

This was in agreement with the hypothesis of neurogenic inflammation that had been proposed earlier. According to this hypothesis, neurovascular interactions in the dura mater are associated with the activation of trigeminal neurons, which results in migraine pain^21^. Inflammatory and vasodilatory neuropeptides such as substance P and calcitonin gene-related peptide (CGRP) are believed to be released onto dural blood vessels, causing vasodilation, plasma protein extravasation (PPE) and associated endothelial cell changes^4^,^8^,^10^,^28^,^29^.

Despite the usefulness that acutely triggering PPE in animal models has had with respect to the characterization of drugs interrupting an ongoing migraine attack, the approach has been falling short in several aspects. First, neurokinin receptor antagonists that fully blocked PPE in these models were not clinically effective for migraine treatment^30^. Second, to our knowledge there is only one case report of intracranial extravasation associated with a migraine attack^31^ while dural PPE has not been demonstrated in human migraineurs otherwise. Even though PPE may thus not be correlated necessarily with migraine pathology in humans, it is interpreted as an indicator for migraine-like pathology and associated changes of the dural endothelium in animal models.

A major discrepancy in studies investigating mCPP-induced PPE has been the fact that 5-HT_2B_ receptor antagonists, which potently prevent the acute mCPP-induced PPE in guinea pigs, require several weeks before their prophylactic action becomes apparent in humans^14^. Here, we observed that mCPP induces PPE only in guinea pigs, while mice and rats did not display a similar effect without pretreatment. Dural neurogenic PPE could, however, be triggered in rodents by electrical stimulation of the trigeminal ganglion^4^,^5^,^6^. Thus, the difference between guinea pigs and rats or mice must reside in mCPP-dependent processes upstream to the activation of trigeminal nerve endings.

It is tempting to speculate that the underlying biochemical or cellular differences between guinea pigs and mice or rats may be of relevance to conditions that predispose migraineurs to develop migraine attacks. In humans, mCPP and NO donors have been shown to induce attacks in migraineurs while subjects without a history of migraine did not react to these compounds, or, for the NO donor nitroglycerin, reported milder headache without a delayed headache phase^26^,^27^,^32^,^33^. All results from animal models must be carefully interpreted, but the observations discussed above indicate that the pathology may reside upstream of the activation of trigeminal afferents, but downstream of the activation of 5-HT_2B_ receptors and, because of the effect of NO donors, cannot be related to the receptor itself.

mCPP is an agonist at both 5-HT_2B_ and 5-HT_2C_ receptors^24^. The selective 5-HT_2B_ receptor antagonists LY202146^6^ or BF-1^16^ blocked the mCPP-induced dural PPE in guinea pigs, whereas LY310898, a specific antagonist for 5-HT_2A_ and 5-HT_2C_ receptors did not prevent dural plasma extravasation^6^. Further evidence that PPE is induced via activation of 5-HT_2B_ receptors stems from the observation that the 5-HT_2B_ receptor-selective agonist BW 723C686 also induced dural neurogenic PPE in guinea pigs, and this response was also blocked by BF-1^16^. These results provide convincing evidence that 5-HT_2B_ receptors are the primary target of mCPP in triggering dural neurogenic inflammation in guinea pigs. The 5-HT_2B_-dependent process, induced by mCPP or BW 723C86, seems to occur prior to the self-reinforcement of NO release that causes dural neurogenic inflammation. This is due to the fact that the NOS inhibitor L-NAME blocked the mCPP-induced PPE in guinea pig and mice, while LY202146 did not block dural PPE after electrical stimulation of the trigeminal ganglion in the guinea pig. Sumatriptan potently blocked mCPP-induced PPE as well as PPE induced by electrical stimulation^6^,^13^. These results are consistent with clinical data illustrating prophylactic efficacy of 5-HT_2B_ receptor antagonists that do not, however, interrupt an ongoing migraine attack^34^.

In contrast to guinea pigs, both mCPP and BW 723C86 failed to induce dural neurogenic PPE in rats or mice in this study. This indicates low susceptibility of these species to triggering the 5-HT_2B_-dependent process that leads to the onset of dural neurogenic inflammation. From animal models of pulmonary hypertension, it is known that chronic hypoxia treatment increased muscularization of blood vessels in the lung^22^. This effect can be inhibited by the application of 5-HT_2B_ receptor antagonists during hypoxia treatment, and 5-HT_2B_ receptor knockout mice did not reveal hypoxia-induced hypermuscularization^23^. The enhanced muscularization was accompanied by elevated expression of 5-HT_2B_ receptors in mouse lung^23^. 5-HT_2B_ receptors are mostly expressed in the vascular endothelium, even though low expression in vascular smooth muscle cells cannot be excluded^18^. We felt tempted to speculate that 5-HT_2B_ receptor-induced blood vessel modulation, seen in terms of hypermuscularization in the lung, may also occur in the dura mater during hypoxia. This speculation was strengthened by the association of migraine and hypoxia: Epidemiological analyses indicated that populations living at high altitudes develop an increased prevalence to migraine^35^,^36^. In addition, normobaric hypoxia was reported as a trigger factor for migraine headache in migraine patients^37^, and symptoms of acute mountain sickness entail symptoms of migraine^38^,^39^. Since furthermore, long-term treatment of migraine patients with 5-HT_2B_ receptor antagonists reduces the frequency and onset of migraine attacks^17^, 5-HT_2B_ receptor-dependent changes in dural blood vessels may be involved.

Based on these thoughts, we kept mice under hypoxic conditions and looked for migraine-associated changes such as the induction of PPE with mCPP. Our data illustrated that four-week chronic hypoxia in a 10% oxygen atmosphere indeed rendered mice susceptible to mCPP-induced dural PPE. The pharmacological characteristics of the induction of dural PPE through mCPP in hypoxic mice were indistinguishable from those in the guinea pig model. These data may be interpreted such that a four-week hypoxia treatment shifted mice from a “non-migraineur” to a “migraineur-like” state, in which mice are sensitive to mCPP. Chronic hypoxia seems to trigger biochemical alterations predisposing to the onset of migraine attacks. According to the arguments above, these alterations may occur downstream from the 5-HT_2B_ receptors but upstream of the activation of trigeminal afferents. The sensitivity towards mCPP developed slowly and lasted for several weeks. It was therefore not an acute effect, but it resolved after several weeks under normoxic conditions. The latter was different from the hypermuscularization in the lung that other groups and we found to remain stable once the hypoxic animals return to a normal atmosphere^25^.

Due to these differences between hypoxia effects in the lung and in the dura mater, we further investigated processes underlying the PPE. To allow investigations by electron microscopy, we used horseradish peroxidase (HRP) as a marker for extravasation. In hypoxic mice, we found HRP-positive vesicles within endothelial cells of venules, capillaries, and arterioles. Interestingly, hypoxia treatment alone triggered slightly elevated baseline transcytosis in all parts of the vasculature when compared to normoxic controls. Even though this difference in baseline was not reflected by elevated PPE, it may nevertheless form the basis for the facilitated induction of PPE. Whether the baseline shift reflects endothelial dysfunction or a proinflammatory state of the endothelium remains to be determined. Hypoxia treatment caused increased haemoglobin levels reflecting elevated haematocrit and higher viscosity of the blood (Supplementary figure S2), but we did not record blood pressure. However, the electron microscopic study did not reveal differences between mice with and without systemic perfusion. Therefore, at least acutely elevated pressure on the vasculature is unlikely to stimulate transcytosis since systemic perfusion exerts a stronger impact on the vasculature than elevated blood pressure.

After mCPP injection, 75–100% of investigated blood vessels in almost all preparations displayed HRP-positive vesicles, indicating that the mCPP-induced PPE is due to increased transcellular transport. There was no indication for a paracellular mechanism of PPE. The tracer HRP was detected in intercellular clefts between endothelial cells, but escape from blood vessels stopped at tight junctions.

During transcellular transport, blood-borne compounds are incorporated into endothelial vesicles at the apical side of endothelial cells and released at the basolateral side. Paracellular transport is due to the disruption of interendothelial junctions, such as tight junctions, and the escape of substances via endothelial clefts. Both mechanisms are well-described events leading to increased permeability and may occur in response to inflammatory stimuli^40^,^41^. In rodent dura mater, they have both been described associated with electrical- or substance P-induced PPE^28^,^29^. Here, we observed that mCPP-induced PPE was associated with increased transcellular permeability in all parts of the vasculature.

If the hypoxic mouse may prove useful as a model for migraine prophylaxis, it must respond to pharmaceutical compounds known to act as migraine prophylactic agents in humans. Daily injections of the mice during hypoxia either with the 5-HT_2B_ receptor antagonist BF-1 or with the NOS-inhibitor L-NAME completely blocked the development of the susceptibility to mCPP-induced PPE. This illustrates a necessary involvement of 5-HT_2B_ receptors and NOS activation in this process.

Higher doses of BF-1 and L-NAME were chosen in chronic than in acute blocking experiments, since intraperitoneally injected compounds are absorbed more slowly when compared with intravenous application. At the high concentration, BF-1 may also block dural 5-HT_2A_ receptors. However, 5-HT_2A_ receptor antagonists did not influence acute mCPP-induced PPE^6^ and did not seem to be involved in migraine prophylaxis^21^,^42^. However, additional pharmacological experiments are required to rigorously exclude participation of 5-HT_2A_ receptors.

5-HT_2B_ receptors and NOS may be involved in two independent aspects in this migraine model: First, the acute effect, triggered by mCPP, might involve activation of the 5-HT_2B_ receptor, NO release, and trigeminal nerve activation, consequently leading to the previously described self-reinforcing mechanism. The same acute effect might equally well be produced by any other agent releasing NO in the endothelium of dural blood vessels, such as histamine as described by García-Cardeña *et al.*^43^.

Second, the chronic hypoxia-induced effect, which led to the facilitation of mCPP-induced PPE, depended on sustained activity of 5-HT_2B_ receptors and NOS. The mechanism how hypoxia treatment caused migraine-like sensitivity remains to be determined. It can, however, not be explained by elevation upregulation of 5-HT_2B_ receptor or NOS RNA expression since quantitative PCR did not reveal hypoxia induced differences in expression levels (data not shown). The role of NOS in migraine pathogenesis has been widely investigated, but its role in the chronic, hypoxia-dependent aspects of this model remains to be investigated further. Interestingly, while specific iNOS inhibitors have not been efficacious in migraine prophylaxis, the efficacy of specific nNOS inhibitors is currently tested^14^,^44^.

The fact that 5-HT_2B_ receptors may be involved in migraine in two independent ways, a chronic and an acute way, explains the apparent discrepancy of the acute action of 5-HT_2B_ receptor agonists in the guinea pig model. The same process is likely to occur in the induction of PPE by mCPP in the hypoxic mouse. This acute process is different, however, from the chronic 5-HT_2B_ receptor-dependent process occurring during hypoxia. We speculate that it is the chronic effect of 5-HT_2B_ antagonists that is experienced clinically when such compounds decrease the frequency and severity of migraine attacks after several weeks of intake^14^.

In addition to NO release, receptor activation may trigger other pathways involved in this process. Most studies investigating the coupling of this receptor type were carried out in heterologous expression systems. Only few investigators studied primary cells, and native 5-HT_2B_ receptor signaling still remains elusive. Studies in heterologous expression systems suggested phosphatidylinositol (PI) hydrolysis, presumably via activation of phospholipase C^45^,^46^ as well as calcium mobilization^47^. In primary cells that endogenously expressed 5-HT_2B_ receptors, such as rat astrocytes primary culture or primary human uterine smooth muscle cells, 5-HT_2B_ receptor activation resulted in calcium mobilization^48^ and PI hydrolysis^49^. Calcium mobilization was independent of PI hydrolysis and sensitive to ryanodine in human primary pulmonary artery endothelial cells that endogenously express 5-HT_2B_ receptors^50^.

In rat stomach fundus and thus in native tissue, 5-HT_2B_ receptor activation was coupled to intracellular calcium increase via voltage-gated channels and from intracellular stores with subsequent protein kinase C activation that was independent of PI hydrolysis^51^.

5-HT_2B_ receptor activation also induced Ras and extracellular signal-regulated kinases (ERK) in mouse fibroblast LMTK- cells transfected with murine 5-HT_2B_ receptors. Through this pathway, 5-HT triggered proliferation and foci formation^52^. The variety of 5-HT_2B_-related signalling cascades suggests that pathways different from NOS activation may be involved and their contribution remains to be investigated in this murine model.

In conclusion, the data presented here indicate that chronic hypoxia treatment renders the mouse dura mater sensitive to mCPP-induced neurogenic PPE involving enhanced transcytosis. Endogenously occurring, chronic 5-HT_2B_ receptor activation is a necessary requirement in this process and can be blocked by daily application of 5-HT_2B_ antagonists. The underlying molecular mechanism of this effect may explain the prophylactic antimigraine effect of these compounds. Investigating 5-HT_2B_ receptor-dependent biochemical changes developing during chronic hypoxia in the mouse dura mater may thus constitute a valuable tool for unravelling the pathological nature of migraine.

## Methods

### Animals

Male and female NMRI-mice (*Mus musculus*), age 8–16 weeks, descended from our own breeding facility or were obtained from Janvier LABS (France). PPE experiments were performed with female mice since we found that due to higher blood viscosity systemic perfusion of hypoxic males was more tedious. As migraine is more prevalent in women^53^, it appeared reasonable to use females for PPE experiments. All animal care and experimental procedures were performed in accordance with EU animal welfare protection laws and regulations. The protocols were approved by the committee on ethics of animal experiments of the state North Rhine-Westphalia. All efforts were taken to minimize animal suffering.

### Chemicals

1-(3-Chlorophenyl)piperazine hydrochloride (mCPP), 1-[5-(thiophen-2-ylmethoxy)-1H-indol-3-yl]propan-2-amine (BW 723C86), 1-[3-(2-dimethylaminoethyl)-1H-indol-5-yl]-N-methylmethanesulfonamide succinate (sumatriptan), Nω-Nitro-L-arginine methyl ester hydrochloride (LNAME), Evans Blue and horseradish peroxidase (HRP) were purchased from Sigma-Aldrich GmbH (Germany). 4-(6-ethoxy-1-methoxythioxanthen-9-ylidene)-1-methyl piperidine maleate (BF-1) was synthetized by Biofrontera Bioscience GmbH at Pharm-Eco Laboratories, Inc. Devens (USA). Saline (0.9%) was used as vehicle for mCPP, L-NAME, HRP and Evans Blue. DMSO in a maximal concentration of 0.1% diluted in saline was used as vehicle for BW 723C86, sumatriptan and BF-1. DMSO (0.1% in saline) by itself did not induce PPE (data not shown). Evans Blue was diluted in saline and filtered with 0.2 μm filter. All test compounds were freshly prepared for each experiment. All doses are expressed per kg bodyweight (BW).

### Hypoxia and normoxia treatment

Normobaric hypoxia chambers maintained hypoxic conditions (10% O_2_ and 90% N_2_) with a 180–200l/h flow, delivered by a membrane pump. Oxygen concentrations were monitored by oxygen sensors. Soda lime was distributed at the bottom of the chambers to regulate CO_2_ levels and calcium chloride (CaCl_2_) was disposed in pillars in the corners of the chambers to regulate humidity. Chambers were opened three times per week for animal care and replacement of soda lime and CaCl_2_. They were opened each day in case of daily pharmaceutical treatment (see below). Hypoxia treatment had various durations of 1 week to 8 weeks with or without subsequent maintenance at normoxic conditions. Under normoxic conditions, animals were kept in the same room but outside the chambers at 21% O_2_ until they were used for experiments. Food and water during hypoxia and normoxia were disposed *ad libitum* and animals were kept under a constant light-dark cycle.

### PPE induction and measurement

Animals were anaesthetized by inhalation of Isoflurane (3–4%), delivered through respiratory masks. Branches of the *Venae jugularis* were exposed for intravenous (i.v.) applications. After careful preparation of the skin, the vena jugularis is easily accessible and big enough for reliable injection. Applications were made the in following order: Compounds that potentially block PPE (L-NAME 10 and 100 μg/kg, sumatriptan 1 and 10 μg/kg, BF-1 1 μg/kg or vehicle) were given intravenously 2 min prior to mCPP or BW 723C86. Compounds that potentially induce a PPE, mCPP and BW 723C86 (0.01 to 1 μg/kg) or vehicle, were applied intravenously. After 2 min, Evans Blue (100 mg/kg) was administered intravenously. After 15 min, animals were euthanized by transcardial perfusion with PBS (0.01 M, pH 7.4). A standardized area of the dura mater (the part of the dura mater that is covering the parietal calvaria) was prepared from the skull and incubated in 100 μl formamide at 22 °C, 500 rpm overnight. The probes were centrifuged (1 min, 14,000 rpm, 22 °C) and photometric measurement (OD 600) of the supernatant was performed. The extent of PPE is given by intensity of OD 600 in the supernatant. Data were normalized to vehicle control.

### Pharmacological intervention during hypoxia treatment

For the pharmacological intervention during hypoxia, the chambers were opened daily and mice received intraperitoneal (i.p.) injections of BF-1/L-NAME or the corresponding vehicle starting from day one of hypoxia. The last application was made on the 28^th^ day of hypoxia. Then, mice recovered in the same room but outside the chambers under normoxic conditions (21% O_2_). After 7 days of normoxic recovery, the experimental procedure of inducing and measuring the PPE was performed as described above. The procedure for “3-days-application” varied so that, mice only received intraperitoneal injection of BF-1/ L-NAME or vehicle on the last three days of hypoxia (day 26-day 28).

### Immunohistological detection of muscularization in dura mater and lung

For investigating the effect in the murine dura mater, animals were anaesthetized with Isoflurane (3–4%). Transcardial perfusion with PBS was followed by perfusion with paraformaldehyde solution (4%, pH 7.4, PFA). Half skull preparations were postfixed in PFA overnight and incubated in ethanol (70%, 2 h, RT). The dura mater was carefully detached from the skull. Whole mount preparations were immediately used for immunohistochemical staining as described for lung sections (as described below, but α-SMA, 1:16,000 diluted in PBS, 8 °C, overnight, ab5694, Abcam, United Kingdom was used). For investigating effects in the murine lung, animals were anaesthetized with Ketamine (0.5%)/Xylazine (0.5%) instead of inhalation anaesthesia due to tracheal dissection. The trachea was dissected and the respiratory tract was filled with PBS to avoid collapse of the lungs during thoracic opening. The lung was perfused with PBS followed by perfusion with PFA. The left lobe was dissected and postfixed in PFA overnight. Tissues were embedded in paraffin and histological sections of 7 μm were prepared.

Lung preparations were stained with a monoclonal antibody against alpha-smooth muscle actin (α-SMA, 1:16,000 diluted in PBS, 8 °C, overnight, A5228, Sigma-Aldrich, Germany). A biotin-linked secondary anti-mouse antibody (1:300 diluted in PBS, RT, 45 min, Vector Larboratories, Germany) was used, followed by incubation with avidin-biotin-complex (1:1:100 diluted in PBS, RT, 45 min, Vector Laboratories, Germany). 3,3′-Diaminobenzidine tetrahydrochloride-dihydrate solution (DAB, 0.07%, Sigma-Aldrich, Germany, diluted in 0.1 M PB, pH 7.4) was used for detection. Lung sections were dehydrated and embedded in Euparal (VWR International, Germany). Dura mater whole mount preparations were carefully stretched on slides and embedded in Immomount (Thermo Scientific, USA). Preparations were photographed by Axioskop 2 microscope (Zeiss, Germany).

To quantify the muscularization in lung, the number of muscularized blood vessels of a diameter 25–50 μm was counted per mm^2^ lung tissue. The murine dura mater tissue is a thin collagenous sheet with spare blood vessels and it was not thus possible to prepare tissue sections that would contain a quantifiable amount of blood vessels. Thus, the length of dural muscularized arterioles of a diameter ≥20 μm was measured in whole mount preparations. To be able to quantify the muscularization level in comparable areas, a coordinate system was developed by BM, using the transverse sinus (as horizontal axis) and sagittal sinus (as vertical axis). An additional horizontal axis was drawn through the pineal gland that often remained attached at the dura mater after the removal of the brain. For quantification, a 7.5 mm^2^ area at the junction of the horizontal and vertical axis was chosen.

### Electron microscopy

Mice were processed as described above, but subsequent to 1 μg/kg (n = 4 hypoxic mice) or vehicle (n = 4 normoxic and n = 2 hypoxic mice), horseradish peroxidase (HRP, 200 mg/kg) was administered intravenously. Animals were perfused with PBS, pH 7.4, followed by perfusion with PFA (4%), glutaraldehyde (2.5%, pH 7.4). For comparison, we also used unperfused tissues that were postfixed overnight. Probes were incubated in a cacodylatbuffer (0.1 M, pH 7.4, 3 × 5 min) and the dura mater was then removed from the skull. For detection, 0.07% DAB-solution (Sigma-Aldrich, Germany, diluted in PB) was used. Probes were incubated in osmiumtetroxide (1% in cacodylatbuffer, 1 h) and then embedded in epoxy resign. Probes were stretched between two aclar foils. Ultrathin sections of one randomly selected part of each preparation were investigated by electron microscopy (Phillips EM 410, Phillips, Netherlands). Photographs of all available blood vessel structures within the specimen were taken by plate negative camera (up to 31,000× magnification; Kodak Electron Microscopy Film 4489, 6.5 × 9 cm). Negatives were developed and scanned (Epson Perfection V500; approximately 3600 × 2800 pixel; 8 bit; 1200 dpi) for investigation. Blood vessel types were characterized by cellular composition and diameter of the vessel lumen. Arterioles revealed a continuous endothelial cell layer and a smooth muscle cell layer, separated by the basement membrane. Capillaries (diameter <10 μm) and venules (diameter >10 μm) both revealed a continuous layer of endothelial cells and displayed a discontinuous layer of pericytes. Blood vessel photographs were then evaluated as positive or negative, with regard to HRP-positive structures in endothelial cells. The blindfolded experimenter performed evaluation. The percentage of dural blood vessels that expressed HRP-positive vesicles in the endothelium is shown for part of the vasculature for each individual.

### Statistics and figure preparation

Figures were prepared using Corel Draw Graphic Suite X6 (version 16). If adequate, only sections of electron microscopic photographs were selected for presentation without changing the resolution. Quantitative data are presented as mean ± SEM. For PPE experiments, data was normalized to the corresponding vehicle control for hypoxic and normoxic mice. To be able to compare hypoxia vs. normoxia treatment in experiments investigating the muscularization of blood vessels in lung and dura mater, the data was normalized to the normoxic controls. Statistics were done using SigmaPlot 12.5. To compare two groups, Rank Sum Test was performed. To compare several groups that differed in several treatment parameters, multiple pairwise comparison (Rank Sum Test) with alpha correction was performed (p = 0.05 divided by the number of performed pairwise comparisons) instead of ANOVA. To compare several groups that differed in only one treatment parameter (e.g. Fig. 2), One-Way ANOVA and Posthoc test (Bonferroni t-test) was performed.

## Additional Information

**How to cite this article**: Hunfeld, A. *et al.* Hypoxia facilitates neurogenic dural plasma protein extravasation in mice: a novel animal model for migraine pathophysiology. *Sci. Rep.* **5**, 17845; doi: 10.1038/srep17845 (2015).
